# Impact of respiratory care training and family support using telemedicine on tracheostomized children admitted with respiratory infection after discharge

**DOI:** 10.1186/s12887-023-04455-7

**Published:** 2023-12-11

**Authors:** Prakarn Tovichien, Nuntiya Khaowsibsam, Bararee Choursamran, Pawinee Charoensittisup, Apinya Palamit, Kanokporn Udomittipong

**Affiliations:** Division of Pulmonology, Department of Pediatrics, Faculty of Medicine Siriraj Hospital, Mahidol University, 2 Prannok Road, Bangkok Noi, Bangkok, 10700 Thailand

**Keywords:** Respiratory care training, Family support, Telemedicine, Tracheostomized children, Respiratory infection

## Abstract

**Objective:**

Children with tracheostomies usually require a long hospital stay, high healthcare costs and caregiver burden. With the help of telemedicine, this study attempted to determine how home respiratory care training and family support affected admission days, admission costs, ICU admission rates, and caregivers’ confidence.

**Methods:**

We enrolled children with tracheostomies who were admitted between 2020 and 2022 with respiratory infections. Before discharge, we evaluated the knowledge and skills of the caregivers and gave them practice in home respiratory care while providing them with structured feedback using a checklist, a peer-to-peer mentor assignment, a virtual home visit, teleeducation, and teleconsultation via a mobile application. We compared the admission days, admission costs, and ICU admission rates one year following the program with the historical control one year earlier.

**Results:**

Forty-eight children with tracheostomies were enrolled. Thirteen percent of those had a 1-year readmission. The median [IQR] number of admission days decreased from 55 [15–140] to 6 [4–17] days (*p* value < 0.001). The median [IQR] admission costs decreased from 300,759 [97,032 – 1,132,323] to 33,367 [17,898—164,951] baht (*p* value < 0.001). The ICU admission rates decreased from 43.8% to 2.1% (*p* value < 0.001). Immediately after the program, caregivers’ confidence increased from 47.9% to 85.5% (*p* value < 0.001).

**Conclusions:**

This respiratory care training and telehealth program decreased admission days, admission costs, and ICU admission rates for children with tracheostomies admitted with respiratory infections. The confidence of caregivers was also increased immediately after the program.

## Background

Tracheostomized children are at increased risk of bacterial tracheostomy-associated respiratory tract infections (bTARTIs), such as ventilator-associated pneumonia (VAP), pneumonia, aspiration pneumonia and tracheobronchitis [1, 2]. Tracheostomy bypasses the natural protective barrier of the upper airway, allowing bacteria to enter the lower respiratory tract easily. Recurrent bTARTIs in these children result in frequent hospitalizations, long admission days, high cost and health care utilization, including ICU admission [2, 3].

All these burdens may be reduced by structured home respiratory care programs, which require skills training of caregivers, such as stromal dressing, suctioning, proper cleaning and disinfection of medical devices, especially in our context, where home nursing care is not included in national health coverage. Therefore, proper evaluation, training and family support from specialized teams are imperative. We designed a multidisciplinary family-centered respiratory care team not only to evaluate and train caregivers in home respiratory care programs but also to support families after discharge by providing them with teleeducation and teleconsultation via mobile applications that they can use for text messages, video calls and education materials. We could also send them medicine and medical devices in response to their request from this telemedicine. This program aimed to reduce the bTARTI rate in tracheostomized children, reduce hospital burdens, including unscheduled admission, admission days, cost of admission and ICU admission rate, which affect both family and hospital burdens, make caregivers feel better prepared and support them while caring for their children at home after discharge.

The goal of this study is to evaluate this family-centered care coordination program and how it affected burdens from bTARTIs, including admission days, cost of admission, ICU admission rate and caregivers’ confidence.

## Methods

This study recruited tracheostomized children less than 18 years old who were hospitalized at Siriraj Hospital, our tertiary care university affiliated hospital in Bangkok, Thailand, with bTARTIs between 2020 and 2022. We excluded children who wished to be followed at other centers after discharge.

A dedicated group of pediatric pulmonologists, pediatricians, nurses, physical therapists, technicians and social workers inspired and directed the development of a pediatric respiratory care team that aimed to innovate a structured home respiratory care program, training curriculum for caregivers, video teaching and education resources to ensure a safe transition home and minimize both caregiver and hospital burden.

The training program included a daily schedule of home respiratory care, such as stoma dressing, suction techniques, chest physiotherapy, emergency tracheostomy tube change, skills for basic life support and disinfection of medical devices. Caregivers practiced continuously under close supervision at bedside by the child’s nurse with additional structured feedback using a checklist. We also added and followed our workflow of discharge planning, peer-to-peer mentor assignments with other families who can share their experiences, easy understanding training videos for teleeducation, virtual home visits and teleconsultation using mobile applications after the child is discharged home.

For each included patient, we collected their demographic data. The outcomes were prospectively followed one year after this multidisciplinary home respiratory care program and compared with historical control one year before the program. The number of admission days, cost of admission and ICU admission rate were used for comparative analysis. All participants had their tracheostomy tube changed at the hospital following the appointment every 2 months, as before this program. Therefore, all the outcome variables were recorded. No oral antibiotics, promotility agents or home care programs from professional personnel services were used during the one-year period after discharge.

We also assessed caregivers’ confidence using a visual analogue scale at the initial evaluation, immediately at the end of the training program and at the re-evaluation 3 and 6 months later. We compared caregivers’ confidence scores before and after the training program and between the re-evaluation and immediately at the end of training.

Categorical variables were reported with absolute numbers and percentages. Continuous variables were reported using medians with interquartile range (IQR). Between-period differences were tested using Wilcoxon’s signed-rank test (continuous variables) or Fisher’s exact test (categorical variables). Statistical analysis was performed using SPSS Statistics 25 (IBM), and a *p* value < 0.05 was considered statistically significant. This study was approved by the Siriraj Institutional Review Board (COA no. Si 615/2020).

## Results

During the study period, 48 tracheostomized children were admitted to Siriraj Hospital due to bTARTIs and were included. Forty-two children (87.5%) were admitted with pneumonia, 2 children (4.1%) were admitted with bacterial tracheitis and 1 child (2.0%) was admitted with infected bronchiectasis. The most common positive culture from tracheal suction was *Pseudomonas aeruginosa* (62.5%). The median [IQR] length of stay was 27 [7–133] days. The median [IQR] age of the subjects was 4 [1-10] years. They were 62.5% male. Thirty-one children (64.6%) underwent tracheostomy for ventilator dependence. Twelve children (25.0%) underwent tracheostomy for upper airway causes. Five children (10.4%) underwent tracheostomy for secretion drainage. Table 1 details the baseline characteristics of the subjects included.

**Table 1:** Demographic data (*n* = 48)

Demographic data	
Age (year)	4 [1–10]
Male	30 (62.5%)
Age at tracheostomy (month)	4 [1–15]
Reason for tracheostomy	
Ventilator dependence	31 (64.6%)
Upper airway obstruction	12 (25.0%)
Secretion drainage	5 (10.4%)
Comorbidities	
Neurological diseases	27 (56.3%)
Chronic lung diseases	11 (22.9%)
Craniofacial anomalies	10 (20.8%)
Gastroesophageal reflux diseases	12 (25.0%)
Tracheal granulation	10 (20.8%)
Tracheostomy type	
Silicone tube	46 (95.8%)
Silver tube	2 (4.2%)
Un-cuffed tube	40 (83.3%)
Cuffed tube	8 (16.7%)
Respiratory support	
Room air	26 (54.2%)
Oxygen collar	7 (14.5%)
Home ventilator	15 (31.3%)
Education of caregivers	
Primary school	8 (16.7%)
High school	27 (56.2%)
More than bachelor's degree	13 (27.1%)
Admission diagnosis	
Infected stomal granulation	5 (10.4%)
Bacterial pneumonia	42 (87.5%)
Bacterial tracheitis	2 (4.1%)
Infected bronchiectasis	1 (2.0%)
Length of stay (days)	27 [7–133]
Intensive care unit admission	20 (41.6%)

Data presented as median [IQR] or number (percent)

In the 1-year cohort after discharge, only 6 children (12.5%) were readmitted. Comparing outcomes one year after with historical control one year before this program, the median [IQR] of admission days decreased from 55 [15–140)] to 6 [4-17] days (*p* value < 0.001). The median [IQR] cost of admission decreased from 300,759 [97,032–1,132,323] to 33,367 [17,898–164,951] baht (*p* value < 0.001). The ICU admission rate decreased from 43.8% to 2.1% (*p* value < 0.001). All heath care utilities one year after the program were significantly reduced from one year before the program, as shown in Table 2. None of them underwent decannulation during this 1-year period.

**Table 2:** Health care utility in 1-year period before and after program

	1-year before	1-year after	*p*-value
Admission days	55 [15-140]	6 [4-17]	<0.001
Cost of admission (baht)	300,759 [97,032-1,132,323]	33,367 [17,898-164,951]	<0.001
ICU admission rate	21 (43.8%)	1 (2.1%)	<0.001

Data presented as median [IQR] or number (percent)

*Abbreviations*: *ICU* Intensive care unit

Most of the main caregivers were mothers (57.9%). The mean (SD) age of caregivers was 39.5 (10.8) years. Seventeen percent of them graduated from primary school, 56.2% of them graduated from high school, and 27.1% of them had more than a bachelor's degree.

The mean (SD) caregivers’ confidence score was 61.7% (22.0) at the initial evaluation, 87.0% (14.6) immediately at the end of the training program, 91.7% (10.3) at the re-evaluation 3 months later and 99.0% (2.8) at the re-evaluation 6 months later. Caregivers’ confidence was significantly improved after the training (*p* value < 0.001) and was persistently higher than baseline in the re-evaluation at 3 and 6 months later (*p* value < 0.001).

## Discussion

Tracheostomized children are at a greater risk of respiratory infections because the tracheostomy tube eliminates the protection provided by the nasal cavity, including filtration, humidification, and warming of the inspired air. It not only creates a direct entrance for pathogens into the lower respiratory tract but also causes a local inflammatory reaction that increases the risk of infection [4]. Furthermore, tracheostomized children may be chronically colonized with bacteria and have impaired airway clearance so that they have high incidence rates of bacterial tracheostomy–associated respiratory tract infections (bTARTIs), such as bacterial pneumonia and tracheobronchitis [2, 5].

Common colonizing bacteria include *Pseudomonas aeruginosa*, *Staphylococcus aureus*, *Morganella morganii*, *Klebsiella pneumoniae*, *Stenotrophomonas spp*. and other gram-negative species [6, 7]. This colonization can increase the risk of infection, usually by the same pathogen. However, some studies also suggest that bTARTIs in tracheostomized children do not occur from colonizing bacteria but from exposure to other bacteria [6]. We should differentiate bTARTIs from bacterial colonization using clinical symptoms of fever, new-onset purulent tracheal discharge and dyspnea in the absence of other causes. Tracheal aspiration for sputum cultures and CXR are helpful in management, but positive cultures alone are insufficient to make the diagnosis [8].

Tracheostomy is an independent risk factor for bTARTIs, resulting in frequent hospitalizations [1–3], ventilator-associated pneumonia (VAP) [9, 10], weaning difficulty, increased exposure to broad-spectrum antibiotics, promoting microbial resistance, increased healthcare costs [8] and contributing to morbidity and mortality [11, 12].

Previous studies have demonstrated that *P. aeruginosa* is a common respiratory isolate in pediatric subjects after tracheostomy [13, 14], as in our study. This result complements recent studies revealing an association between *P aeruginosa* isolation and poorer outcomes, including readmission for bTARTI [14], ICU admission and ventilation support [15]. Moreover, subjects may require longer hospitalization for intravenous antibiotics due to the limited enteral alternatives for *P. aeruginosa* treatment.

There were some quality improvement projects organizing a trained expert committee of pediatric tracheostomy care to evaluate the discharge process, develop an education protocol, and review and revise education materials, which revealed inconsistent results.

Well et al [11] revealed that their program decreased the 7-day and 30-day unplanned readmission rates. In contrast, Zhu et al [2] revealed that there was no decrease in the overall or tracheostomy-related readmission rate but a significant decrease in tracheostomy-related wound complications. Children discharged home were significantly more likely to be admitted for a tracheostomy-related complication than subjects discharged to an advanced care facility.

Graf et al [16] showed that caregivers of children with new tracheostomies needed a median of 14 days (range 5–110 days) to successfully complete a tracheostomy education program. In this program, caregivers viewed education videos, read internet handouts, observed, and then actively participated in suctioning and tracheostomy care, attended cardiopulmonary resuscitation (CPR) classes and had 24-h rooming with home equipment. Discharge occurred a median of 6.5 days (range 0–71 days) after education was completed. Common impediments to completing the education program included social issues, intercurrent illness and/or language barriers. Impediments to discharge included patient's intercurrent illnesses, social issues, and unavailability of home nursing. Tolomeo et al [17] revealed that their program decreased the time required by parents to achieve proficiency in the care of a technology-dependent infant and the length of stay for these infants. Baker et al. [18] revealed that their program for tracheostomized children using home ventilators decreased the length of stay (LOS) and costs without a negative impact on patient safety.

This research aims to outline a quality improvement project directed toward establishing a structured pediatric home respiratory care program with the goal of improving the quality of care these children and caregivers receive, as in previous studies. However, our context is different: home nursing care services are not covered in our national health coverage, and most caregivers must perform all home respiratory care for their children on their own.

Proper home respiratory care programs potentially diminish unscheduled admission, admission days, cost of admission, ICU admission rate and caregiver burden. The underlying hypothesis behind this program is that improving caregiver training regarding optimal home respiratory care may assist secretion drainage and decrease the infection rate and healthcare utilization. Twenty percent of children in our cohort had tracheal granulation. Hence, improper tracheostomy care might contribute to their infection. Moreover, this study added to the innovation of tele-education and tele-monitoring in response to the COVID-19 pandemic. Although caregivers would have their questions and concerns post discharge, they could also address more promptly using tele-consultation via text message or video call and would not require frequent unscheduled visits and hospital admissions, thereby improving the quality of life for these children. In these small cohorts of children and caregivers, our program appeared to significantly improve the quality of care they received.

Identifying risk factors associated with revisit rates may help our team to identify subjects who will benefit from close supervision and further interventions. From a previous study, cerebral palsy and GERD were associated with infections in children with tracheostomy [1]. In Russell et al.’s study, the median LOS in bTARTIs was 4 days, which was much lower than that in our study, but the 30-day revisit rate was 30.5%, which was higher than that in our cohort. They found that ventilator-dependent subjects, age less than 12 months old or at least 4 complex chronic conditions (CCCs) were at highest risk for both longer LOS and 30-day revisit after discharge for bTARTIs [3], which was different from our study, which could not find any risk factors due to the limitation of low rate of 1-year readmission. However, the majority of subjects in our study also had neurological diseases which confirmed the high burden of bTARTIs in this population. In Russell et al.’s study, neuromuscular and metabolic conditions showed the strongest associations with increased LOS and increased odds of 30-day revisit. Renal and cardiovascular conditions were associated only with increased odds of 30-day revisit [3]. Public insurance, ICU admission, acute mechanical ventilation and empirical anti-Pseudomonas antibiotics were associated with longer LOS but not with increased revisit odds. We plan to perform further study to identify whether those variables impact our cohort.

There are also limitations of this study. The sample size was quite small. However, we could spend time training all the participants closely. The same personnel trained each skill for all participants, which promoted their proficiency. The assessment of knowledge and skills of caregivers in this study make the educator know the gap of our training and provide us the opportunities for improvement of our training program including emergency simulation practice and teleconsultation.

Caregivers admired our training for easy understanding. The caregivers were objectively graded on their basic knowledge about tracheostomy and their competency in providing proper tracheostomy care to their children, including tasks such as dressing, suctioning, and initial management for emergency problems like bleeding through the tracheostomy tube or tracheostomy tube occlusion. We also debriefed and rectified the misunderstandings and pitfalls of caregivers. Subsequently, we reevaluated their knowledge and skills using a competency checklist before discharge. Their major complaint was a load of detailed information, and they truly liked tele-education programs, including education videos and summarized articles that they could review after discharge. They also loved to perform virtual home visits using mobile applications to show us how proficiently they performed home respiratory care programs for their children, told their concerns and asked about problems at their homes, which was much more convenient than visiting us at the hospital. This project represents pilot work to study the initial effectiveness of our program that combined physicians, nurses, social workers, and caregivers to create a coordinated program that addressed the needs of caregivers and support the family during hospitalization with bTARTIs and after they transitioned home. Tele-education and tele-consultation are important tools for family support, especially during the COVID-19 pandemic.

Providers can use this information when counseling families about possible adverse outcomes after tracheotomy and guide them in developing home respiratory care training programs and family support using tele-education, tele-consultation and peer-to-peer mentor assignments with other families to improve home respiratory care programs and minimize LOS and cost of admission, which reflect both hospital and family burden.

In conclusion, our quality improvement project to develop a comprehensive home respiratory care training program and family-centered support using telehealth for tracheostomized children admitted with bTARTIs suggests a model for reducing admission days, cost of admission and ICU admission rate and increasing caregivers’ confidence, which is worth further development.

## Data Availability

The datasets used and/or analyzed during the current study are available from the corresponding author upon reasonable request.
